# Sexual Quality of Life, Sexual Knowledge, and Attitudes of Older Adults on the Example of Inhabitants Over 60s of Bialystok, Poland

**DOI:** 10.3389/fpsyg.2018.00483

**Published:** 2018-04-11

**Authors:** Mateusz Cybulski, Lukasz Cybulski, Elzbieta Krajewska-Kulak, Magda Orzechowska, Urszula Cwalina, Marek Jasinski

**Affiliations:** ^1^Department of Integrated Medical Care, Faculty of Health Sciences, Medical University of Bialystok, Bialystok, Poland; ^2^National Security Student, Faculty of Social Sciences, University of Warmia and Mazury in Olsztyn, Olsztyn, Poland; ^3^Department of Statistics and Medical Informatics, Faculty of Health Sciences, Medical University of Bialystok, Bialystok, Poland; ^4^Department of Psychology, Non-State Higher Pedagogical School in Bialystok, Bialystok, Poland

**Keywords:** ASKAS, attitudes, knowledge, elderly, older adults, sexual quality of life, SQOL

## Abstract

**Introduction:** Aging has a strong influence on the quality of relationships and sexual functioning, but in itself does not cause a lack of sexual desire.

**Objectives:** The aim of this study was to assess the quality of sexual life and define sexual knowledge and attitudes of older people on the example of residents of Bialystok, Poland at the age of 60 and over.

**Methods:** The study included 170 people, inhabitants of Bialystok, Poland aged over 60: 85 students of the University of Healthy Senior and the University of Psychogeriatric Prophylaxis and 85 students of the University of the Third Age. The study used three standardized psychometric scales: Sexual Quality of Life Questionnaire-Male (SQoL-M), Sexual Quality of Life Questionnaire-Female (SQoL-F), and Aging Sexual Knowledge and Attitudes Scale (ASKAS).

**Results:** The overall mean score for the ASKAS scale for knowledge was 65.21 ± 12.32 and for attitudes −124.65 ± 22.00. The overall mean SQOL score was 62.92 ± 18.18. Taking into account the gender of the respondents, the knowledge of men on sexuality of seniors was at the level of 63.48 ± 12.63, while in the female group −65.74 ± 12.23. The attitudes of men on sexuality of seniors was at the level of 128.80 ± 21.56, while in the female group −123.38 ± 22.05. Satisfaction with sex life among men (72.36 ± 27.49) was significantly higher than among women (60.02 ± 12.88).

**Discussion:** The seniors were characterized by moderate knowledge and attitudes to sexuality of older people and the average level of sexual satisfaction. There was no significant relationship between knowledge on sexuality and sexual satisfaction in the study groups, and there was a positive correlation between attitudes toward sexuality and the satisfaction of sex life outside the group of men. In addition, a significant positive relationship was found between attitudes toward sexuality and sexual satisfaction. In order to improve the knowledge of senior citizens about sexuality of old age and to overcome the taboos that are prevalent in this topic, a structured training should be provided in this field. Such training should be carried out by specialists in the field of sexology. It is desirable to conduct in-depth studies in the assessment of knowledge, attitudes, and quality of sexual life in a larger research group, in order to get results for the population of the whole country.

## Introduction

The aging of a population poses a significant challenge to public health, both in social and health terms. By 2020, more than one million Poles will be 90 years old, and by 2035 more than 25% of Poles will be 65 years old and older. In 2060, Poland will have one of the oldest populations in Europe (Mossakowska et al., 2012). In 2014, in the Podlasie voivodship lived 225,901 people (19% of the total) over 60s. The percentage of older people in the Podlasie voivodship increases year by year. Among the elderly population, the majority are women (68.1%). The Podlasie voivodship is one of the oldest voivodships in Poland. In this region, women live longest in the country (Central Statistical Office in Poland, 2014).

Aging has a strong impact on the quality of relationships and sexual functioning, whereas, human sexuality is one of the key elements of life quality and may be significant in maintaining proper human relationships and the feeling of being an integral part of the society (Zanni et al., 2003). It does not cease to exist in old age—it still remains important for older people and can improve their overall quality of life (Hajjar and Kamel, 2003), however, it is often threatened, especially among the residents of nursing homes (Shuttleworth et al., 2010). Another factor demotivating elderly to express their sexuality is negative stereotypes and myths prevailing in the society about sex life of seniors, and describing them as deprived of sexual interests (Bauer et al., 2013). Sexual identity is connected with self-esteem and, if rejected, it can have a detrimental effect on self-esteem, social relationships, and mental health of seniors (Doll, 2013).

Adequate sexual life is important for well-being in adulthood, in particular among older adults, and the subjectively perceived sexual quality of life has various connections with many functional domains (Forbes et al., 2017). Although, there is an inadequate level of research on sexual quality of life in older adulthood, some Western studies have shown that older adults show less sexual satisfaction than younger adults (Araujo et al., 2004; Lindau and Gavrilova, 2010; Field et al., 2013). Preliminary evidence also suggests a special association for the elderly people between a higher sexual quality of life and better physical health in the USA (Lindau and Gavrilova, 2010)—as well as greater satisfaction with life in Israel (Woloski-Wruble et al., 2010). However, sexuality in aging remains a largely unexplored area of research (Koh and Sewell, 2015). In addition to determining which time-related factors affect the apparent negative relationship between age and sexual quality of life, it is important to identify specific mechanisms that can take into account differences between individuals and individual changes in sequal quality of life over time. The most likely explanation for age-related changes in subjective sexual quality of life appears to be a simultaneous decrease in other sexuality domains with age. For example, population studies in the USA and the United Kingdom have shown that the frequency and likelihood of engaging in sexual activity are now negatively related to age from middle age (Schick et al., 2010; Field et al., 2013; Thomas et al., 2015; Lee et al., 2016), in particular women (Lindau et al., 2007).

There is limited research examining Polish elderly people sexual quality of life, knowledge and attitudes toward their sexuality. Moreover, there are currently no recommendations concerning how older men or women can manage or overcome their sexual problems, and there are no studies that allow a comparison of sexual behaviors across many different countries. This knowledge could be used as a stepping stone to the enhancement of sexual health in older adults. No comprehensive, nationally representative, population-based data are available to inform physicians' understanding of the sexual norms and problems of older adults. Finally, sexual aging can have harmful consequences for older people, both through individualized sexual stigma and sexual problems, as well as restrictive policies and procedures that affect sexual expression and health. Ultimately, the goal is for older people to feel safe to achieve sexual well-being in a society that understands their needs and supports sexual expression throughout their lifetime.

In connection with the above, the aim of this study was to assess the quality of sexual life, define sexual knowledge and attitudes of older people on the example of inhabitants of Bialystok, Poland at the age of 60 and over and assess the relationship between the quality of sexual life of older people and their sexual knowledge and attitudes. We hypothesize that women will have a lower sexual quality of life than men and sexual quality of life, sexual knowledge, and attitudes of older adults will be negative.

## Materials and methods

### Participants

The study was conducted in two groups:

- group I—students of the University of Healthy Senior (UHS) and the University of Psychogeriatric Prophylaxis (UPP) [85 people, including 67 women (78.82%) and 18 men (21.18%)], carried out at the Faculty of Health Sciences of the Medical University of Bialystok;
- group II—students of the University of the Third Age in Bialystok (UTA) [85 persons, including 63 women (74.12%) and 22 men (25.88%)].

The study included 170 people in total, residents of Bialystok, at the age of 60 and over −130 women (76.47%) and 40 men (23.53%). In the group from UHS and UPP the youngest respondent was 60 years old, while the oldest −78 years old. The median age was 67.22 years. Among the students of UTA the median age was 65.72 years—the youngest respondent was 60 years old, and the oldest −85 years old. The median age of the whole study group was 66.47. The socio-demographic characteristics of the sample are shown in Table 1.

**Table 1:** Socio-demographic characteristics of the sample.

**Feature**		**UHS/UPP**		**UTA**		**Total**	
		***n***	**%**	***n***	**%**	***n***	**%**
Gender	Women	67	78.82	63	74.12	130	76.47
	Men	18	21.18	22	25.88	40	23.53
Age	≤ 70 years	70	82.35	76	89.41	146	85.88
	≥ 71 years	15	17.65	9	10.59	24	14.12
Marital status	Married	39	45.88	46	54.12	85	50.00
	Widowed	27	31.76	23	27.06	50	29.41
	Single	2	2.35	1	1.18	3	1.76
	Divorced	14	16.47	13	15.29	27	15.88
	Separated	3	3.53	2	2.35	5	2.94
Financial situation	Very good	5	5.88	5	5.88	10	5.88
	Good	33	38.82	34	40.00	67	39.41
	Rather good	17	20.00	20	23.53	37	21.76
	Average	30	35.29	24	28.24	54	31.76
	Rather bad	0	0.00	1	1.18	1	0.59
	Bad	0	0.00	1	1.18	1	0.59
Education	Higher education	37	43.53	40	47.06	77	45.29
	Secondary	40	47.06	36	42.35	76	44.71
	Technical	6	7.06	2	2.35	8	4.71
	Vocational	2	2.35	3	3.53	5	2.94
	Primary	0	0.00	4	4.71	4	2.35
Total		85	100.00	85	100.00	170	100.00

### Data collection

Besides the age and place of residence, an additional criterion for inclusion in the study was to give a written consent for participation in the study. Each respondent could withdraw from it at any time.

The selection of the respondents was intentional. The authors assumed collecting at least 150 fully completed questionnaires, 75 in each study group. A greater number of the research tool copies was distributed, but not all of the questionnaires were returned to the authors of the study. We distributed 150 copies of questionnaires among the students of the UHS and UPP, and 350 copies among the participants of the UTA. The number of returned questionnaires was 119 in group I (response rate −79.3%), and 131 in group II (response rate −37.4%), but not all returned surveys were complete. After analyzing all the returned questionnaires authors received 85 complete questionnaires form each group.

### Measures

The study design was cross-sectional. The study used three standardized psychometric scales:

- Sexual Quality of Life Questionnaire-Male (SQoL-M);
- Sexual Quality of Life Questionnaire-Female (SQoL-F);
- Aging Sexual Knowledge and Attitudes Scale (ASKAS).

The Sexual Quality of Life-Male (SQOL-M) is a short questionnaire that specifically assesses the relationship between male sexual dysfunction and quality of life. It contains 11 items, each with a 6-point Likert scale ranging from “completely agree” to “completely disagree.” The items are scored from 1 to 6 points (worst to best)—Completely Agree = 1 to Completely Disagree = 6. The total score can range from 11 to 66 points. The higher the score, the greater the quality of life (Abraham et al., 2008).

The Sexual Quality of Life-Female (SQOL-F) is a short questionnaire that specifically assesses the relationship between female sexual dysfunction and quality of life. The SQOL-F questionnaire is a specific and self-reporting instrument that focuses on sexual self-esteem, emotional issues and relationship issues. It consists of 18 items, rated using a six-point scale (completely agree to completely disagree). The total score can range from 18 to 108 points. Higher scores indicate better female sexual quality of life (Symonds et al., 2005).

For easier comparison raw scores of SQOL-M and SQOL-F will be transformed using a standardized scale of 0 to 100, using the following formula (Symonds et al., 2005; Abraham et al., 2008):

$$\text{Scale score }=\;\frac{\text{the sum of components}-\text{the lowest possible score}}{\text{possible raw score range}}\;*\;100$$

The Aging Sexual Knowledge and Attitudes Scale (ASKAS) represents an instrument for the assessment of sex cognition related to aging (White, 1982). The ASKAS is designed for older people, people who work with them, and any group of people who have an impact on them (e.g., families, volunteers working with older people). The questions are designed to measure sexual attitudes and sexual knowledge using items dealing with age-related changes (and lack of changes) in sexuality and sexual context in older people. The ASKAS consists of 61 questions, 35 of which are “true”, “false,” and “don't know,” and 26 have a 7-point Likert scale indicating the extent of agreement or disagreement with the statements (White, 1982). The true-false questions measure older peoples' knowledge on sexuality, while the agree-disagree Likert scale questions measure their attitudes toward sexuality. The lower the score the greater the knowledge. For the attitude items, lower scores indicate a permissive attitude. White (1982) provided data on the reliability of the instrument, with alpha reliability results ranging between 0.76 and 0.93, and test-retest results ranging between 0.72 and 0.92.

The respondents received hard copies of questionnaires, which they completed at home after a detailed discussion with the members of the research team. The discussion focused on the characterization of each research tools and a detailed explanation of how to complete it.

### Procedure and ethical considerations

The study was performed from April to July 2017 in accordance with the recommendations, and was reviewed and approved by the Bioethics Committee of the Medical University in Bialystok (statute no. R-I-002/35/2017). All subjects gave the written informed consent in accordance with the Declaration of Helsinki.

### Statistical analysis

The following descriptive statistics were used to describe the quantitative variables: arithmetic mean, standard deviation, and median. The normalization of numerical variables was assessed using the Shapiro-Wilk test. For variables with normal distribution, the *t*-Student test was used to compare the two groups. In case of non-observance of normal distribution, the *U* Mann-Whitney test was used. The Pearson's correlation coefficient was used to evaluate the relationship between quantitative variables. A statistical analysis was performed using a statistical package, STATISTICA 12. It was assumed that statistically significant results were at the level of *p* < 0.05.

## Results

### Sexual knowledge of the respondents

The overall mean score for the ASKAS scale for knowledge was 65.21 ± 12.32. In the group of students of UHS and UPP the score for knowledge on sexuality of older people was 61.89 ± 10.70 and in the UTA group −68.52 ± 13.00. The optimum number of points was 35, but none of the respondents got such a value. The lowest score received in the study was 39 points in the UHS and UPP group and 43 in the UTA group. According to the interpretation of the scale, the lower the point value for knowledge, the greater the knowledge. This means that UHS and UPP students had greater knowledge. There were statistically significant differences between the knowledge on sexuality of older people in the group of UHS and UPP participants, and the UTA students. The knowledge questions are presented in Table 2.

**Table 2:** ASKAS—knowledge questions (White, 1982).

Sexual activity in aged persons is often dangerous to their health.
Males over the age of 65 typically take longer to attain an erection of their penis than do younger males.
Males over the age of 65 usually experience a reduction in intensity of orgasm relative to younger males.
The firmness of erection in aged males if often less than that of younger persons.
The older female (65+ years of age) has reduced vaginal lubrication secretion relative to younger females.
The aged female takes longer to achieve adequate vaginal lubrication relative to younger females.
The older female may experience painful intercourse due to reduced elasticity of the vagina and reduced vaginal lubrication.
Sexuality is typically a lifelong need.
Sexual behavior in older people (65+) increases the risk of heart attack.
Most males over the age of 65 are unable to engage in sexual intercourse.
The relatively most sexually active younger people tend to become the relatively most sexually active older people.
There is evidence that sexual activity in older persons has beneficial physical effects on the participants.
Sexual activity may be psychologically beneficial to older person participants.
Most older females are sexually unresponsive.
The sex urge typically increases with age in males over 65.
Prescription drugs may alter a person's sex drive.
Females, after menopause, have a physiological-induced need for sexual activity.
Basically, changes with advanced age (65+) in sexuality involve a slowing of response time rather than a reduction of interest in sex.
Older males typically experience a reduced need to ejaculate and hence may maintain an erection of the penis for a longer time than younger males.
Older males and females cannot act as sex partners as both need younger partners for stimulation.
The most common determinant of the frequency of sexual activity in older couples is the interest or lack of interest of the husband in a sexual relationship with his wife.
Barbiturates, tranquilizers, and alcohol may lower the sexual arousal levels of aged persons and interfere with sexual responsiveness.
Sexual disinterest in aged persons may be a reflection of a psychological state of depression.
There is a decrease in frequency of sexual activity with older age in males.
There is a greater decrease in male sexuality with age than there is in female sexuality.
Heavy consumption of cigarettes may diminish sexual desire.
An important factor in the maintenance of sexual responsiveness in the aging male is the consistency of sexual activity throughout his life.
Fear of the inability to perform sexually may bring about an inability to perform sexually in older males.
The ending of sexual activity in old age is most likely and primarily due to social and psychological causes rather then biological and physical causes.
Excessive masturbation may bring about an early onset of mental confusion and dementia in the aged.
There is an inevitable loss of sexual satisfaction in post-menopausal women.
Secondary impotence (or non-physiologically caused) increases in males over the age of 60 relative to young males.
Impotence in aged males may literally be effectively treated and cured in many instances.
In the absence of severe physical disability males and females may maintain sexual interest and activity well into their 80s and 90s.
Masturbation in older males and females has beneficial effects on the maintenance of sexual responsiveness.

### Sexual attitudes of the respondents

The overall mean score for the ASKAS scale for attitudes was 124.65 ± 22.00. In the group of UHS and UPP students, the attitude toward sexuality of seniors was at the level of 127.42 ± 23.02 and in the UTA group −121.88 ± 20.69. The lowest scores received in the study were 68 points in the group of UHS and UPP students, and 60 in the UTA group. According to the interpretation of the scale, lower scores for attitudes indicate greater acceptance of the attitudes in sexuality of older people. This means that students of UTA were characterized by more open-minded attitudes, as opposed to the level of their knowledge. The attitudes questions are presented in Table 3.

**Table 3:** ASKAS—attitudes questions (White, 1982).

Aged people have little interest in sexuality.
An aged person who shows sexual interest brings disgrace to himself/herself.
Institutions, such as nursing homes, ought not to encourage or support sexual activity of any sort in their residents.
Male and female residents of nursing homes ought to live on separate floors or separate wings of the nursing home.
Nursing homes have no obligation to provide adequate privacy for residents who desire to be alone, either by themselves or as a couple.
As one becomes older interest in sexuality inevitably disappears.
If a relative of mine, living in a nursing home, was to have a sexual relationship with another resident I would complain to the management.
If a relative of mine, living in a nursing home, was to have a sexual relationship with another resident I would move my relative from this institution.
If a relative of mine, living in a nursing home, was to have a sexual relationship with another resident I would stay out of it, as it is not my concern.
If I knew that a particular nursing home permitted and supported sexual activity in residents who desired such, I would not place a relative in that nursing home.
It is immoral for older persons to engage in recreational sex.
I would like to know more about the changes in sexual functioning in older years.
I feel I know all I need to know about sexuality in the aged.
I would complain to the management if I knew of sexual activity between any residents of a nursing home.
I would support sex education courses for aged residents of nursing homes.
I would support sex education courses for the staff of nursing homes.
Masturbation is an acceptable sexual activity for older males.
Masturbation is an acceptable sexual activity for older females.
Institutions, such as nursing homes, ought to provide large enough beds for couples who desire such to sleep together.
Staff of nursing homes ought to be trained or educated with regard to sexuality in the aged and/or disabled.
Residents of nursing homes ought not to engage in sexual activity of any sort.
Institutions, such as nursing homes, should provide opportunities for the social interaction of men and women.
Masturbation is harmful and ought to be avoided.
Institutions, such as nursing homes, should provide privacy such as to allow residents to engage in sexual behavior without fear of intrusion or observation.
If family members object to a widowed relative engaging in sexual relationships with another resident of a nursing home, it is the obligation of the management and staff to make certain that such sexual activity is prevented.
Sexual relations outside of the context of marriage are always wrong.

### Sexual quality of life of the respondents

The overall mean SQOL score was 62.92 ± 18.18. In the group of UHS and UPP students sexual satisfaction was 61.88 ± 18.61 and in the UTA group −63.97 ± 17.68. The highest scores obtained in the study were 100 points in the UHS and UPP students group and 99 points in the UTA group. This means that the UTA students were characterized by greater satisfaction of sex life, however the differences were not statistically significant.

### Sexual knowledge and sexual attitudes in terms of gender of the respondents

Taking into account the gender of the respondents, the knowledge of men on sexuality of seniors was at the level of 63.48 ± 12.63, while in the female group −65.74 ± 12.23. The lowest scores received in the study were 41 points for men and 39 points for women. This means that men were characterized by a higher level of knowledge. The reverse situation was reported in case of attitudes toward sexuality of older people. More positive attitudes were observed in women, however, no statistically significant differences were found. Whereas, in case of satisfaction of the respondents with their sex life, the satisfaction in the group of men was significantly greater than in women (Table 4).

**Table 4:** Comparison of the results for SQOL and ASKAS in terms of origin group and gender of respondents.

**Variable**	**UHS & UPP (*****n*** = **85)**			**UTA (*****n*** = **85)**			***p***	**Men (*****n*** = **40)**			**Women (*****n*** = **130)**			***p***
	**$\bar{x}$**	**SD**	**Me**	**$\bar{x}$**	**SD**	**Me**		**$\bar{x}$**	**SD**	**Me**	**$\bar{x}$**	**SD**	**Me**	
Age	67.22	4.46	66.00	65.72	4.76	65.00	0.020*	67.15	5.93	66.00	66.26	4.20	66.00	0.747
SQOL	61.88	18.61	61.11	63.97	17.68	64.44	0.454	72.36	27.49	85.45	60.02	12.88	60.56	<0.001*
ASKAS_knowledge	61.89	10.70	62.00	68.52	13.00	67.00	<0.001*	63.48	12.63	62.00	65.74	12.23	65.00	0.311
ASKAS_attitudes	127.42	23.02	126.00	121.88	20.69	119.00	0.101	128.80	21.56	127.50	123.38	22.05	121.50	0.173

UHS, University of Healthy Senior; UPP, University of Psychogeriatric Prophylaxis; UTA, University of the Third Age; SQOL, Sexual Quality of Life; ASKAS, Aging Sexuality Knowledge and Attitudes Scale; $\bar{x}$, mean; SD, standard deviation; Me, median; p, p-value; *statistically significant value.

### Correlations between the quality of life and sexual knowledge and attitudes

The Pearson's correlations between satisfaction with sex life and knowledge and attitudes to sexuality of older people were performed. There was no significant relationship between the knowledge on sexuality and the satisfaction with sexuality in any of the subgroups. There was a positive correlation between attitudes toward sexuality of older people and sexual satisfaction. The correlation between knowledge and attitudes toward sexuality of the elderly and sexual satisfaction was not demonstrated in the male group (*p* = 0.105 and *p* = 0.094). The higher the scores for attitudes toward sexuality of older people, the higher the satisfaction with sexual life of the respondents (Table 5).

**Table 5:** Pearson correlation between sexual satisfaction with life and knowledge and attitudes in the sexuality of older persons.

	**UHS & UPP (*n* = 85)**		**UTA (*n* = 85)**		**Total (*n* = 170)**		**Men (*n* = 40)**		**Women (*n* = 130)**	
	***r***	***p***	***r***	***p***	***r***	***p***	***r***	***p***	***r***	***p***
ASKAS_knowledge	0.003	0.978	0.144	0.188	0.091	0.239	0.260	0.105	0.037	0.672
ASKAS_attitudes	0.331	0.002^*^	0.240	0.027^*^	0.278	<0.001^*^	0.268	0.094	0.287	0.001^*^

UHS, University of Healthy Senior; UPP, University of Psychogeriatric Prophylaxis; UTA, University of the Third Age; ASKAS, Aging Sexuality Knowledge and Attitudes Scale; r, Pearson correlation coefficient; p, p-value;

* *statistically significant value*.

## Discussion

Most of the available publications focus primarily on the published studies on the perception of sexuality of older people by medical personnel, i.e., doctors and nurses (Ali, 2004; Gott et al., 2004a,b; Roach, 2004; Gunderson et al., 2005; Ward et al., 2005; Burd et al., 2006), and to a lesser extent they include studies carried out in the senior population, concerning their sexual behaviors, sexual activity, and knowledge in this field (Guan, 2004; Henderson et al., 2004; Rosen et al., 2004; Moreira et al., 2005). So far, this is the first study of that kind, including the population of older people in Poland.

It is commonly agreed upon among experts and the literature on ageism and sexual aging suggest that elderly people continue to be viewed according to stereotypes of incompetence and asexuality (Weeks, 2002; Cuddy et al., 2005; Huffstetler, 2006). However, when reviewing empirical research on attitudes toward aging sexuality, many studies report moderately permissive and positive attitudes, when examining specific age groups (Steinke, 1994; Hillman and Stricker, 1996; Spector and Fremeth, 1996; Bouman et al., 2006; Allen et al., 2009).

In our study, the ASKAS's mean score for knowledge was 65.21 ± 12.32, and for attitudes −124.65 ± 22.00. The obtained results did not confirm the hypothesis that sexual knowledge and attitudes of older adults will be negative and they were the most positive in comparison with the results of other studies. In a Steinke's study (Steinke, 1994) the respondents showed moderate knowledge and attitudes, without statistically significant differences between men and women. In a study of Chen et al. (2017) the mean score for knowledge on sexuality of seniors (ASKAS) was 22.8 on a scale of 13 to 31, while the mean score for attitudes toward sexuality of older people (ASKAS) was 68.79 of 182 possible points. Here, it is worth mentioning another method of interpretation of results with the use of this scale by Chen et al. (2017), which was not consistent with the principles established by White (1982), the author of the ASKAS scale (the scores in questions about attitudes assessment have not been reversed). Nurses from a study by Mahieu et al. (2016) were characterized by a moderate level of knowledge and showed a rather positive attitude toward sexuality of older people. In addition, this study showed that a higher level of knowledge on sexuality of older people is associated with a more positive attitude toward sexuality in later life. In a study by Bauer et al. (2013) the average total number of ASKAS points in a pre-test was 54.21 ± 9.924, while the mean total score for ASKAS in the subsequent test was 50.92 ± 12.897. In a study by Langer-Most and Langer (2010), including 141 gynecologists, the median score for knowledge in the ASKAS scale was 49 ± 8, and the mean score for attitudes was 81 ± 17. In the above mentioned study, as opposed to our own research, there was no correlation between the respondents' knowledge and their attitudes (Langer-Most and Langer, 2010). A study by Dogan et al. (2008) showed that Turkish doctors of various specialties had limited knowledge on sexuality of older people, but their attitudes toward the sexuality of seniors were positive. Similarly to our study, the examined women had more negative attitudes to sexuality of seniors than men. In addition, the authors showed that physicians with longer seniority had a higher level of knowledge than younger physicians, but their attitudes toward older people's sexuality were similar (Dogan et al., 2008). A study by Snyder and Zweig (2010) including 100 students of medicine and psychology, showed that both groups did not have sufficient knowledge about sexuality of older people. A study by Quinn-Krach and Van Hoozer (1988) including nursing students showed that greater knowledge was associated with more positive attitudes. Age was also significantly correlated both with positive attitudes and a higher level of knowledge on sexuality of seniors. Older students had more positive attitudes toward seniors and had a higher level of knowledge on the sexuality of older people than younger respondents.

In our study, the overall mean score for SQOL was 62.92 ± 18.18, with 72.36 ± 27.49 for men, and 60.02 ± 12.88 for women. The results confirmed the hypothesis that that women will have a lower sexual quality of life than men, which is consistent with past research for example from the USA (Carpenter et al., 2009; Lindau and Gavrilova, 2010; Forbes et al., 2017). In addition, the study showed that the sexual satisfaction of the elderly was at a moderate level, which was confirmed by the results of other authors' research.

Strizzi et al. (2015), who carried out a study including patients with traumatic brain injury and healthy subjects showed that the level of sexual satisfaction among patients was 56.54 ± 16.56 and in the control group −84.64 ± 13.00. In a study by Andersson et al. (2012) it was proven that sexually active women had a higher mean score in the SQOL-F scale (76.26 ± 24.19) than non-active women (44.13 ± 25.31). Among people with physical disabilities in Ghana, satisfaction with sex life was 50.9 ± 20.9—among men it was 45.7 ± 22.9 and among women −57.7 ± 15.8 (Owiredu et al., 2015). In a group of Moroccan patients with chronic lower back pain, satisfaction with sex life was 44.6 ± 17.4 (Bahouq et al., 2013). In a study by Bernie et al. (2017) satisfaction with sexual life among men was 37.7 ± 3.3 and in the control group 39.2 ± 2.2. The mean scores of participants in a Korean study conducted by Kim and Kang (2015) showed that the evaluation of sexual satisfaction was at the level of 74.25 ± 13.65. In a study by Rogers et al. (2008) SQOL of women in the study group was 69.6 ± 23.1 and 69.2 ± 23.0 in the control group. Golbasi and Erenel (2012) showed that sexual satisfaction among women with reproductive system cancer was 52.50 ± 22.87, including 50.07 ± 22.60 among women over the age of 50 and 50.82 ± 22.42 for married women aged 50 and over. A study of women before and after bariatric surgery found that women before the procedure assessed the quality of sex life at 60.0 and women after the surgery −75.0 (Janik et al., 2015). Similar studies were conducted among men—in the control group, the quality of sexual life was 81.8 and in the group after the surgery −88.6 (Janik et al., 2016).

The results of this study are limited by the small size of the study group, especially a small sample of older men, and the geographic limitation to the respondents from one city. In our opinion, the subject of sexuality of the elderly turned out to be too controversial and embarrassing for the elderly, which was reflected in the low percentage of returned complete questionnaires. This confirmed our assumptions that the study subject is still taboo for Polish seniors, and they are not too eager to express their opinions on the analyzed issues.

In conclusions, the seniors were characterized by moderate knowledge and attitudes to sexuality of older people and the average level of sexual satisfaction. There was no significant relationship between knowledge on sexuality and sexual satisfaction in the study groups, and there was a positive correlation between attitudes toward sexuality and the satisfaction of sex life outside the group of men. In addition, a significant positive relationship was found between attitudes toward sexuality and sexual satisfaction. In order to improve the knowledge of senior citizens about sexuality of old age and to overcome the taboos that are prevalent in this topic, a structured training should be provided in this field. Such training should be carried out by specialists in the field of sexology. It is desirable to conduct in-depth studies in the assessment of knowledge, attitudes and quality of sexual life in a larger research group, in order to get results for the population of the whole country.

## Author contributions

MC and EK-K contributed to the study design, MC and LC contributed to the data collection. All co-authors contributed to data analysis and interpretation. All co-authors contributed to the writing process. The first author took part in the whole process. All authors have read and approved the final manuscript.

### Conflict of interest statement

The authors declare that the research was conducted in the absence of any commercial or financial relationships that could be construed as a potential conflict of interest.
